# An automated compound screening for anti-aging effects on the function of *C. elegans* sensory neurons

**DOI:** 10.1038/s41598-017-09651-x

**Published:** 2017-08-24

**Authors:** Daphne Bazopoulou, Amrita R. Chaudhury, Alexandros Pantazis, Nikos Chronis

**Affiliations:** 1 Department of Mechanical Engineering, University of Michigan, Ann Arbor, MI 48109 United States of America; 2 Department of Biomedical Engineering, University of Michigan, Ann Arbor, MI 48109 United States of America

## Abstract

Discovery of molecular targets or compounds that alter neuronal function can lead to therapeutic advances that ameliorate age-related neurodegenerative pathologies. Currently, there is a lack of *in vivo* screening technologies for the discovery of compounds that affect the age-dependent neuronal physiology. Here, we present a high-throughput, microfluidic-based assay for automated manipulation and on-chip monitoring and analysis of stimulus-evoked calcium responses of intact *C. elegans* at various life stages. First, we successfully applied our technology to quantify the effects of aging and age-related genetic and chemical factors in the calcium transients of the ASH sensory neuron. We then performed a large-scale screen of a library of 107 FDA-approved compounds to identify hits that prevented the age-dependent functional deterioration of ASH. The robust performance of our assay makes it a valuable tool for future high-throughput applications based on *in vivo* functional imaging.

## Introduction

Methods for automated manipulation and *in vivo* imaging of small model organisms such as *C. elegans* have a great potential to revolutionize the drug discovery process^1–4^. Microfluidics can play a pivotal role as they enable process automation, assay standardization and collection of a large number of high quality, user-independent data. A handful of microfluidic platforms have been developed so far that, coupled with powerful optomechanical systems, have allowed serial or parallel immobilization of worms for high resolution imaging^5–8^. By adapting automated image acquisition and data analysis strategies and with the use of genetically expressed fluorescent probes, these platforms have greatly exploited *C. elegans* advantages for *in vivo* genetic and compound screens^9–12^. However, the screens performed so far are merely based on phenotypic analysis of morphology and/or gene expression patterns rather than monitoring functional changes in cellular processes. A significant development towards this direction is the combination of calcium-based functional imaging with microfluidics in a unique and powerful state-of-the-art tool that enables the probing of neuronal activity *in vivo*, from large worm populations^13^.

*C. elegans* with its powerful genetics and a well-defined nervous system has become a prominent model organism for studying *in vivo* the process of aging^14–18^. Studies have revealed several molecular pathways that affect the worm’s lifespan^19^. Genes involved with the insulin/IGF-1 signaling pathway^20^, caloric restriction^21^ and mitochondrial function^22, 23^, enhance environmental stress resistance and significantly increase the worm’s lifespan. Compounds that affect the worm’s lifespan have also been studied. The antidepressant drug mianserin which targets serotonergic signaling was discovered in a large-scale screening to extend the lifespan of *C. elegans* by ~30% through dietary restriction mechanisms^24^. Resveratrol, the plant-derived polyphenol initially shown to activate the catalytic activity of sirtuins^25^, consists another pharmacologic life-extension paradigm not only in *C. elegans* but also in yeast and fruit flies^25–27^. Sirtuins have been shown to mediate some of the beneficial effects of caloric restriction^28^. There is also evidence of a protective role of sirtuins in brain aging and neurodegeneration^29, 30^.

Understanding the physiology of aged neurons is a particularly challenging task, as aging studies need to be performed in a well-controllable environment. A large number of data is also required to obtain statistically significant trends. The discovery of neuron-specific, anti-aging compounds poses an extra challenge as the biochemical and cell-based assays fail to represent the long-lasting accumulative effects of aging. These effects would be ideally represented in physiologically intact animals. Studies in *C. elegans* have shown that, although there is no neuronal loss, a significant deterioration of neuronal structures and function begins as early as early-mid adulthood. Age-dependent morphological changes such as ectopic branching, swelling and lesions of neuronal processes were observed in the mechanosensory, motor and dopaminergic circuits^31–33^. Another study showed by electrophysiological methods that a progressive decline in the function of *C. elegans* motor neurons can be improved pharmacologically^34^. Despite some initial success, the difficulty of manipulating large populations of aging worms and the lack of a robust assay for monitoring their nervous system in a high throughput manner has prevented the wide use of *C.elegans* for anti-aging, drug screening applications. Here, we describe the development of a high-throughput, microfluidic-based platform for functional imaging applications also compatible with multi-well plate architecture. The platform serially loads and immobilizes worms to effectively evoke and record calcium transients from single sensory neurons in a non-invasive fashion. To demonstrate the applicability and throughput of the platform we first monitored stimulus-evoked, calcium responses from the ASH polymodal neuron over the animal’s lifespan. Our analysis showed a progressive age-dependent deterioration of ASH activity. Then, we performed a screen of a customized library of 107 FDA-approved drugs and identified tiagabine, as a compound that ameliorate neuronal activity in aged animals.

## Results

### Multi well-plate compatible platform for automated calcium imaging

**Figure 1:**
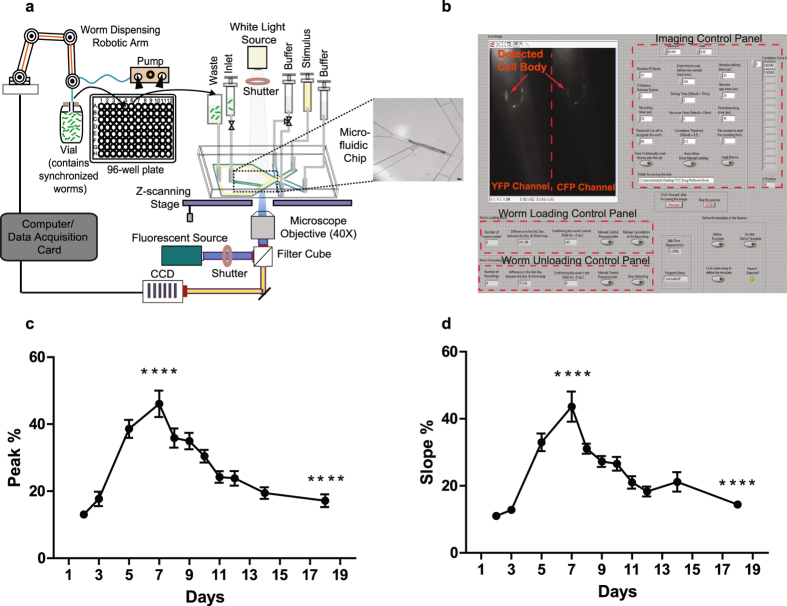
The automated platform and the effects of aging in ASH transients. (**a**) The robotic arm transfers worms from a vial to a 96-well plate and then into the biochip (Scale bar, 20 µm). The entire process is computer-controlled (worm handling, neuronal recognition, stimulation and imaging). (**b**) Snapshot of the graphical user interface (GUI) showing a real-time, fluorescence image of the worm’s nose and the three control panels (worm loading, unloading and imaging) where the user selects the screening parameters. (**c**) Averaged peak [maximum FRET value of the rising phase (see also Supplementary Fig. 2c)] of ASH calcium transients in response a 30 s pulse of 1 M glycerol during adulthood. (**d**) Averaged slope [rate of FRET change during the rising phase (see also Supplementary Fig. 2c)] of ASH calcium transients in response a 30 s pulse of 1 M glycerol during adulthood. Error bars indicate SEM. Between 39 and 58 worms were imaged for each time point in triplicate. ****p < 0.0001, one-way ANOVA followed by *t*-test pairwise analysis to compare Day 7 to Day 3 and Day 18 to Day 7.

The developed platform consists of a multi-well plate-to-biochip interface module and a microfluidic-coupled functional imaging module (Fig. 1a). As part of the first module, a liquid-handling robotic arm transfers worms from a vial to the wells of a multi-well plate and to a microfluidic biochip. This module also incorporates a liquid pump that operates all the liquid withdrawing/dispensing tasks. The imaging module consists of: (i) a microfluidic biochip, termed the ‘olfactory biochip’^35^, specifically designed to manipulate worms in an automated fashion, (ii) an inverted, fluorescent microscope and (iii) a graphical user interface (GUI) (Fig. 1b). The platform operates with only three computer-controlled valves. One valve is designated to deliver the stimulus and two valves serve for worm transferring, loading and unloading.

The biochip incorporates a worm trap that serially immobilizes worms for high-resolution imaging and a 4-flow microfluidic architecture that precisely delivers a chemical stimulus to the worm’s nose (Supplementary Fig. S1). An additional side channel, the ‘flush’ channel, is used to unload trapped worms from the biochip. A compressed air source is used to manipulate worms on-chip by facilitating the liquid flow into the chip. The biochip’s dimensions are optimized to accommodate worms of different sizes/ages, grown either in liquid culture or in agar plates (Supplementary Tables S1 and S2).The platform operates in a fully automated fashion. The robotic system transfers worms into the biochip. Once a worm is loaded into the trap, an image-processing algorithm determines the worm’s head-to-tail orientation by identifying the fluorescently labeled neuron and discards worms entering the trap by tail. After the head-to-tail orientation is confirmed, the neuron is detected and brought into focus by either an edge detection or a correlation-based, template matching algorithm, suitable for younger (larval stages and Day 1 to Day 4 adults) and gravid worms (Day 5 and older adults) respectively (see Methods and Supplementary Note). Then, the stimulus is delivered to the worm’s nose and the neuronal response is recorded. Finally, the worm is flushed out of the biochip by pressurizing the side microfluidic channel and the next worm is loaded. An automated washing step is introduced once the biochip finishes processing a user-defined number of worms. To eliminate clogging of the device, all the solutions used (growth media and flow/stimulation buffers) are filtered before introducing them into the biochip. To dissolve any aggregates, the robotic arm mixes by pipetting the worm solution multiple times before loading the worms in the chip.

The platform’s operation is controlled through a GUI panel. The user can select the wells from the plate to be tested, recording and stimulus delivery times and loading/unloading speed. The front panel also includes controls for imaging parameters such as the camera’s exposure time, cut-off threshold for the recognition of the worm’s entrance, redefinition of the neuronal template, fine-tuning of the autofocus and also the file path for storing the recorded data (see Methods and Supplementary Note).

To monitor neuronal activity, we used the TNXL calcium sensor^36^ and a FRET imaging setup. Following the acquisition and storage of the raw imaging data, an image tracking software extracts the FRET ratio during neuronal stimulation (Supplementary Fig. S2a and b). The software tracks the fluorescent changes and the extracted FRET ratios are compiled in a single excel file and further processed in Matlab to compensate for photobleaching effects. The slope (rate of FRET change) and peak (maximum FRET value) of the rising phase are extracted from the photobleaching-corrected FRET ratios (Supplementary Fig. S2c) and are reported as a percentage change from the baseline over time.

### Interrogation of functional aging of ASH neuron

To test whether the platform’s operation enables the study of biological processes characterized by population variability, we monitored ASH responses as a function of age. We recorded ratiometric responses from hundreds of worms grown in liquid culture in the presence of a 30 s, hyperosmotic (1 M glycerol) stimulus pulse (Supplementary Fig. S3). In this case, the robotic arm was continuously providing worms for imaging from a single vial. We recorded from neurons with average fluorescence intensities, as we empirically found that worms that were too bright or too dim did not elicit any measurable responses. To quantify the stimulus-evoked calcium transients, we obtained the FRET ratio intensity traces and quantified the age-dependent parameters of the rising phase i.e. the peak (total Ca^+2^ entering the cell) and slope (rate of Ca^+2^ change). These metrics represent the magnitude and time response of cell depolarization, respectively. For simplicity, we artificially divided the days of observation during adulthood into an early phase (Day 2 to 5), mid phase (Day 7 to 8) and late phase (>Day 9). We found that the peak and slope of ASH responses rapidly increase during the early phase to reach their maximum at Day 7 of adulthood (mid phase), followed by a gradual decline during late phase (Fig. 1c,d). At Day 18, the ASH responses have already decreased by over 50% compared to the mid phase responses. Mortality slightly affected the population at Day 14 (6%) and reached 15% at Day 18 (data not shown). Dead worms were identified by the lack of movement and pharyngeal pumping while on chip and discarded without recording. In all of the following experiments we did not observe any differences in the mortality rates between control and compound treated or mutant worms within the same day of observation. We did not find any correlation between the stimulus-evoked calcium transients and the pre-stimulation intracellular Ca^+2^ levels (Supplementary Fig. S4). We did however notice that an increased baseline ratio of ASH responses correlates with the maximum Ca^+2^ changes observed (Supplementary Fig. S5a–d) during the mid phase. We also followed the evolution of the expression levels of the FRET sensor during our course of observation. We found no correlation between ASH fluorescence and the peak of Ca^+2^ responses (Supplementary Fig. S6a–d).

The platform is capable of loading and unloading a single worm in ~8–10 s, resulting in a processing speed of 400–450 worms/h, excluding the user-defined imaging time. The processing time is not affected by the worm’s age (Supplementary Table S3). The platform enables processing of worms grown either in liquid or solid media and ranging in size from the early L3 larval stage till Day 5 of adulthood. Based on our observations, Day 5 represents the age at which worms, grown in liquid culture, acquire their maximum size.

### Effects of lifespan-extending factors on ASH function during aging

**Figure 2:**
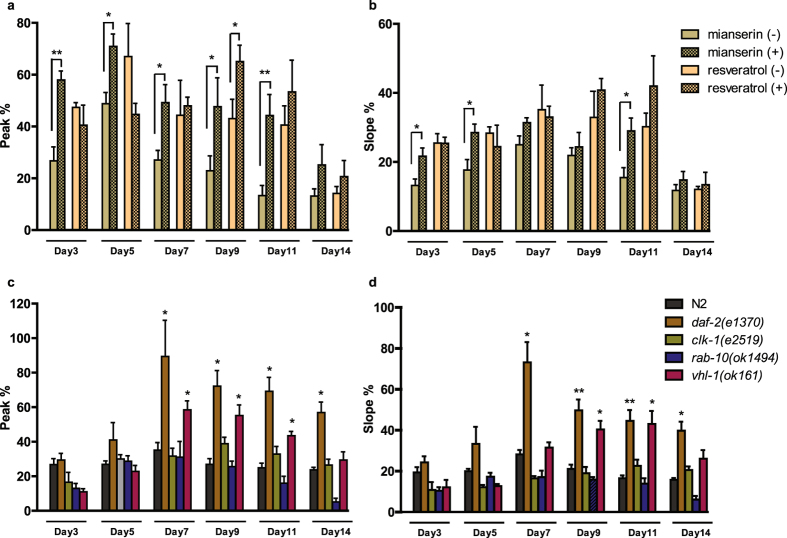
Effects of lifespan-extending factors in ASH transients in response to 1 M glycerol. Bar graphs depict the averaged peak (**a**) and slope (**b**) of ASH responses in aging worms treated with either 50 µM mianserin or 50 µM resveratrol starting at Day 1 of adulthood. Bar graphs depict the averaged peak (**c**) and slope (**d**) of ASH responses from well-characterized long-lived mutants. Error bars indicate SEM. Between 28 and 48 worms were imaged for each time point in triplicate. *p < 0.5, **p < 0.01, two-way ANOVA followed by Bonferroni’s test for multiple comparisons.

The robust performance of the system further allowed us to easily assess the impact of longevity-promoting compounds and gene mutations. First, we tested the impact of mianserin and resveratrol in aging animals grown in liquid media, at concentrations that are known to extend lifespan^24, 37^. Mianserin increased ASH responses at most time points tested starting from the early phase, suggesting a neuromodulatory effect on ASH activity (Fig. 2a,b). This was expected, since mianserin affects neuronal signaling by serotonin^24^ and directly increases the sensitivity of ASH nociceptive responses^38–40^. Interestingly, a recent study found the lifespan-extending effect of mianserin to be mechanistically related to this increase of synaptic transmission^41^. On the other hand, reservatrol had a subtle enhancing effect only at late phase in adulthood, suggesting a potential neuroprotective role on aging ASH (Fig. 2a,b).

Animals with increased lifespan and healthspan due to a mutation in the gene encoding DAF-2 (insulin/IGF-1 receptor)^42, 43^ exhibited an increased ASH activity in both early and late adulthood (Fig. 2c,d). Previous studies have shown that *daf-2* mutation delays age-dependent structural and presynaptic release defects in *C. elegans* touch and motor neurons respectively^31–33^. In agreement with these studies, our results provide further evidence that *daf-2* indeed regulates the progression of functional aging in neurons. However, other mutations that confer to lifespan extension such as *clk-1*, a demethoxyubiquinone hydroxylase required for mitochondrial respiration^44^, *vhl-1* that targets the HIF-1 Hypoxia-Inducible Factor for degradation^45^ and *rab-10*, a Rab-like GTPase that regulates autophagy in response to dietary restriction^46^, did not significantly ameliorate ASH function in aging worms (Fig. 2c,d). Overall, these observations suggest that increased lifespan is not a direct predictor of delayed neuronal decline.

### Screening of FDA-approved compounds and target validation

Taking advantage of the platform’s high-throughput capability, we performed a pilot screen of a large population of compound-treated worms to discover compounds that can restore ASH function during aging. We used a custom-made library of 107 FDA-approved compounds, known to affect the human central nervous system, in a 96 well-plate format (Methods and Supplementary Table S4). The entire screening process was performed automatically and controlled via the GUI. The robot withdrew microliter volumes from a vial containing 15–20 synchronized Day 5 adult worms and distributed them to each well of the 96-well plate. The wells contained 20 µM of each compound, dissolved in 0.4% DMSO. DMSO had no significant effect on ASH responses (Supplementary Fig. S7). Worms were incubated into the well plates for several days. During that period, the robot dispensed a small amount of OP-50 bacteria-contained medium every 36 hours to assure worms are not starving. After the desired aging period, the robot withdrew worms from the well plate and dispensed them into the inlet of the biochip. The biochip serially trapped, exposed and imaged worms recovered from each well. To avoid cross-contamination, the tubing of the robot was cleaned from traces of each compound by dipping the dispensing tip into a cleaning solution. When the biochip finished processing worms from one well, the robot withdrew worms from the next well and loaded them into the biochip. This process continued until worms from all wells were tested.

We chose to initiate the compound treatment in the well plates at Day 5 in order to eliminate any toxic effects of the compounds on worm’s development. The worms after a continuous incubation in the wells, were transferred into the biochip as Day 12 adults, and imaged at a rate of 1 worm/100 s (5 s for worm loading, 40 s pre-stimulation exposure time, 50 s for neuronal stimulation/recording, 5 s for worm unloading). The 40 s pre-stimulation exposure time was used to obtain a stable calcium baseline prior to stimulation (Supplementary Fig. S8), as ASH responds to UV light^40^. At such a throughput rate, we were able to process an entire 96-well plate in ~24 h. Approximately 30% of the worms either entered the trap by tail or had a low ASH fluorescence intensity and were discarded without being recorded. We strictly followed the timed protocol described above to ensure that all worms were maintained for the same amount of time in the biochip prior to and during imaging. Furthermore, for every 9 compound wells we processed, we run worms from a control well to ensure a comparison between treated and untreated worms of exactly the same age.

**Figure 3:**
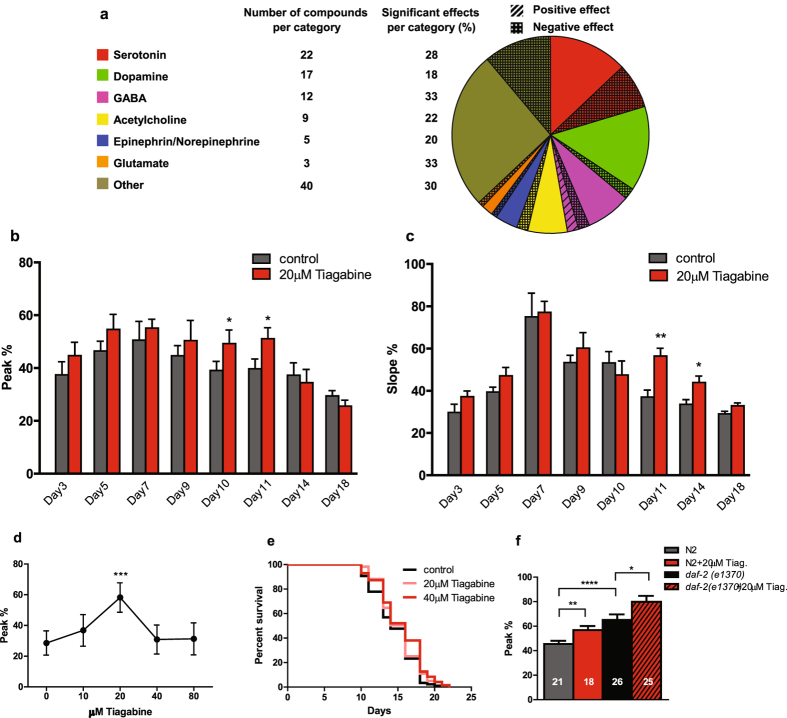
Results from the *in vivo* screen of 107 FDA-approved compounds. (**a**) The pie chart represents the percentage of compounds with a positive, negative or no effect on ASH response. Bar graphs depict the averaged peak (**b**) and slope (**c**) of ASH responses during adulthood after the administration of 20 µM tiagabine in Day 1 adults. Error bars indicate SEM. For (**b**) and (**c**) between 28 and 51 worms were imaged. Experiments were carried out in triplicate. *p < 0.5, **p < 0.01, two-way ANOVA followed by Bonferroni’s test for multiple comparisons (**d**) Dose response curve to various concentrations of tiagabine administered at Day 5 of adulthood. Worms were imaged at Day 12. Error bars indicate SEM. Between 25 and 33 worms were imaged in triplicate. ***p < 0.001, one-way ANOVA followed by *t*-test pairwise analysis to compare to untreated control. (**e**) Lifespan assay at 20 μM and 40 μM of tiagabine. Median survival: control = 14, (n = 86); 20 μM tiagabine = 16, (n = 119); 40 μM tiagabine = 16, (n = 71). Statistical analysis: log-rank (Mantel-Cox) test. f) Bar graph depicting the average peak of ASH responses at Day 12 in *daf-2(e1370)* worms in the presence of 20 µM tiagabine. Error bars indicate SEM. Numbers on bars indicate number of worms imaged in triplicate. *p < 0.5, **p < 0.01, ****p < 0.0001, one-way ANOVA followed by *t*-test pairwise analysis.

Out of the 107 drugs tested (Fig. 3a and Supplementary Table S4), tiagabine and honokiol showed a significant positive effect (Table 1 and Supplementary Fig. S10), with tiagabine provoking the highest, over 50% increase in ASH response. Tiagabine is classified as a GABA reuptake inhibitor^47, 48^ and is used as an antiepileptic drug. Honokiol is a pleiotropic natural product proposed to act on distinct molecular targets including GABA_A_ receptors^49^. To evaluate whether the onset of the treatment with respect to the worm age affects the aging process, we administered 20 μM tiagabine in Day 1 adult worms. In the case of tiagabine, we found that the ASH responses were enhanced in older animals, which was consistent with our screening results (Fig. 3b,c). The lack of a significant positive effect at early phase of adulthood agrees with a protective role of tiagabine against age-dependent neuronal deterioration. The diminished effect at the very end of late phase (Day 14 and Day 18) is likely attributed to the development of drug tolerance due to the prolonged treatment. A dose response curve revealed that higher concentrations did not significantly affect ASH calcium responses when compared to the control (Fig. 3d). Lifespan assays at 20 μM and 40 μM tiagabine did not confer to lifespan extension (Fig. 3e). When tiagabine was administered in long-lived *daf-2* mutants, an additional increase in ASH activity was observed (Fig. 3f), suggesting that tiagabine’s molecular targets act in parallel to the IGF-1/insulin signaling pathway.

**Table 1:** FDA-approved compounds that have a significant effect on ASH activity.

Compound	Molecular Target	*Effect on Neuronal Function vs Control (%)	*p*-value
Tiagabine	GABA transporter 1	55.6	0.002
Honokiol	GABA_A_ receptor/NF-κB/Nitric oxide synthase	46.4	0.008
Dolasteron	Serotonin 5-HT3 receptor	−40.3	0.001
L-694,247	Serotonin 5-HT1D receptor	−37.9	0.001
Levosulpiride	Dopamine D2 receptor	−36.3	0.01
Flumazenil	Benzodiazepine receptor	−44.8	0.0004
Nitroindazole	Nitric oxide synthase	−41.4	0.02
Rolipram	Phosphodiesterase-4	−35.5	0.0008

*The effect on neuronal function as quantified by the percent difference in the peak value of ASH responses with respect to untreated worms of the same age (Day 12 of adulthood). Worms were exposed to drugs starting at Day 5.

Animals exposed to honokiol as Day 1 adults showed only a short term increase in calcium responses at 20 μM concentration (Supplementary Fig. S9a,b and c). This suggests that honokiol does not confer any anti-aging benefits and the improvement on ASH activity, as detected in our screen, was a result of neuromodulation. In fact, higher concentrations of honokiol had a negative in lifespan (Supplementary Fig. S9d). Interestingly, 24 out of the 107 FDA-approved and potentially neuroprotective compounds tested in our preliminary screen had a negative effect in ASH responses (Fig. 3a, Table 1 and Supplementary Fig. S10). This could be due to toxic effects from physiologically high compound concentrations and remains to be investigated in future validation studies.

## Discussion

Current research efforts in the field of aging aim to identify (a) genes and molecular pathways that are involved in the process of aging, and (b) chemical compounds and environmental conditions that could decelerate the aging process. Several obstacles, unique to aging research, are present. Aging is a slowly evolving and life-lasting process, resulting in long experimental procedures. Its stochastic nature requires monitoring a large population of subjects throughout their lifespan, which is an expensive and impractical solution. Finally, current drug discovery approaches are based on molecular or cell-based assays that cannot represent the complex, *in vivo* environment of an aging organism.

The discovery of compounds that preserve the function of neurons in intact organisms opens up a new direction in drug screening methodology. *C. elegans*, due to its small size, transparency, ease of growth and manipulation in liquid cultures is amenable to high throughput protocols including screening assays based on fluorescent imaging^9, 11, 12^. Despite this progress, there are still many barriers to overcome, including high quality, automated functional imaging and analysis. The goal of this work was to develop a platform that would combine monitoring of functional properties of neurons via calcium imaging with automated worm processing protocols. Ultimately, we aimed to interrogate neuronal activity during aging.

Our automated liquid handling robotic system eliminates any labor intensive steps such as multiple transfers of worms for incubation and monitoring as well as the delivery of bacterial food during the long-term incubation. The platform operates at a rate of 1 worm/10 sec, which includes worm loading, target recognition/focusing, and worm unloading with no interruption. The optimized biochip design and operational conditions enables fast immobilization of worms of different ages, ensuring consistent handling for all worms before and during imaging. Moreover, the biochips can be reused several times after a thorough cleaning step. Using our software, we substantially decreased the data acquisition and image analysis time to meet high throughput requirements. At the same time, we improved the quality of imaging by adjusting our detection and auto-focusing algorithms to age-dependent factors such as the intensity and shape of fluorescent objects. Automated data processing also eliminated user’s biases during the extraction of the calcium transients’ metrics (i.e. peak, slope).

Calcium ions generate a multitude of intracellular signals that control key functions in all types of neurons, such as neurotransmitter release and gene transcription^50^. Calcium imaging is currently the most popular, state-of-the-art technique that enables direct measurement of the dynamic calcium flux within neurons upon stimulation^51^. During the development of our platform, we imaged multiple populations of adult *C. elegans* at different stages. The plarform’s robust performance enabled us to monitor and quantify the age-dependent decline in one aspect of ASH function (responses to hyperosmotic stimulus). The maximum calcium transients were observed during mid adulthood, followed by a progressive decrease in older animals. The evolution of ASH responses during aging was independent of the intensity of the calcium sensor. Therefore, and in conjunction with our previous findings^13^, we conclude that monitoring of calcium dynamics, has a great potential for correlating neuronal function with aging. Additional calcium imaging studies in downstream interneurons and behavioral assays can further elucidate the effects of aging in the neuronal circuit of ASH.

We next examined the effects of mutations and known compound treatments (resveratrol, mianserin) that extend *C. elegans* lifespan. Interestingly, with the exceptions of the *daf-2* mutation and the resveratrol treatment, we discovered minimum effects of the other tested conditions on preserving the function of ASH during aging. This indicates that not every genetic or pharmacological lifespan extension paradigm correlates with a delayed onset of neuronal decline.

Finally, the integration of microfluidic technology with multi-well plate architecture enabled us to run a screen for the identification of neuroprotective compounds in aging worm populations. Our customized library contained chemically synthesized compounds (e.g. tiagabine, dolasetron, levosulpiride) and a collection of natural-derived products (e.g. honokiol, epigallocatechin, isoquercetin) that target the central nervous system. The platform operated continuously with no manual intervention and allowed us to process ~1500 aged worms distributed in 120 wells, including the controls, in 48 hours. We anticipated that an anti-aging compound will have a dual action upon stimulus delivery. That would be to improve neuronal sensitivity (increase the peak of the response) and decrease the time constant (increase the slope). We found 2 compounds that fulfill these criteria and increased ASH activity by 2-fold: the chemically synthesized compound tiagabine and the plant-extracted polyphenol honokiol. Further investigation enabled us to discriminate between an improvement against age-dependent functional decline (tiagabine) and a modulatory effect on targets that directly or indirectly send feedback to ASH neuron (honokiol).

Tiagabine is an antiepileptic drug which targets GABAergic signaling in mammals^52^. There is no report of a GABAergic modulation of ASH calcium responses, so far. An indirect effect of tiagabine on ASH function, through other neuromodulatory loops and in aging worms, remains to be determined. Additional experiments to test for anti-aging effects of tiagabine in other non-GABAergic neurons should be carried out in future studies. Moreover, although there was no extension of lifespan under tiagabine treatment, it would be interesting to study other physiological markers for healthspan extension.

*daf-2* mutants, which not only live longer but appear healthier than wild type worms at the same age^19^ have been previously shown to have a reduced functional decay on neuromuscular junctions^34^. Our observations provide additional evidence to propose that *daf-2* mutations result in decelerated, functional decline in the nervous system. Tiagabine further enhanced ASH function in long-live *daf-2* mutants, suggesting that its mechanism of action is independent to the insulin-like signaling pathway.

Overall, we demonstrated that our live animal screening technology is capable of pairing functional imaging with high-quality drug discovery assays analogous to those devised for cell-based protocols. The developed technology is not limited to aging research; it can be useful to researchers that require a large number of *in vivo* imaging data. Examples include probing the activity of chemosensory neurons or neuronal circuits and also applications varying from drug discovery to forward genetics and RNAi screens.

## Methods

### *C. elegans* strains and handling

Standard procedures were followed for *C. elegans* strain maintenance and genetic crosses^53^. The strains used in this project included NKC2311 *micIs211(sra-6p::TN-XL)*, NKC20 *daf-2(e1370);micIs211(sra-6p::TN-XL)*, NKC23 *clk-1(e2519);micIs211(sra-6p::TN-XL)*, NKC19 *rab-10(ok1494);micIs211(sra-6p::TNXL)*, NKC25 *vhl-1(ok161);micIs211(sra-6p::TN-XL)*. ASH activity during aging in mutant strains and in the presence of resveratrol and mianserin was assessed in liquid media [S-complete with pen/step (40 units/ml) and nystatin (100 units/ml)] containing freshly prepared *E. coli* OP50–1 as feeding bacteria (~2 × 10^9^ bacteria mL^−1^). Briefly, worms were synchronized and feeding bacteria were added at the L1 larva stage. To prevent self-fertilization, FuDR was added 42–45 h after the addition of bacteria at 20 ^0^C. For the compound treatments, animals were exposed to 50 µM of resveratrol or mianserin. Stock solutions for these compounds were prepared in H_2_O and DMSO respectively. For the experiment with the mutants, animals were raised at 15 °C [permissive temperature for the *daf-2(e1370)*] and shifted to 20 °C as L4 larvae, to circumvent dauer entry for the *daf-2* mutant. For all experiments the first day of adulthood was considered as Day 1.

### Lifespan assays

Lifespan studies were performed at 20 ^0^C in NGM agar media and in the presence of FuDR. The compounds were administered at the indicated final concentrations before plate pouring. Survival was scored every 2–3 days, and worms were censored if they crawled off the plate, hatched inside, or lost vulva integrity during reproduction. Each experiment included 70–110 worms and was repeated twice.

### Compound library preparation and treatment

The small molecule library consists of the majority of the CNS targeting compounds from the BioFocus NIH Clinical Collection and was obtained from the University of Michigan CCG (Center for Chemical Genomics). The compounds, initially stocked at 5 mM DMSO, were arrayed in single wells of 96-well microtiter plates (#3364; Corning) using the TTP Labtech Mosquito ×1. Plates were prepared in triplicates and were heat-sealed and stored at 20 °C until the day of the screening. Column 11 of each plate was used for arraying intra-plate controls (0.4% DMSO). Columns 1 and 12 and rows A and H contained assay medium only to eliminate the occurrence of edge effects. Each compound was screened at a final concentration of 20 μM in the presence of 0.4% DMSO. Age-synchronized animals were seeded as L1 larvae in liquid medium [S-complete with pen/step (40 units/ml) and nystatin (100 units/ml)] containing freshly prepared *E. coli* OP50–1 as feeding bacteria (~2 × 10^9^ bacteria mL^−1^) and grown at 20 °C. To prevent self-fertilization, FuDR was added 42–45 h after seeding. Beginning at Day 5 of adulthood (~165 h after seeding), 15–20 animals contained in 100 µl liquid culture were transferred in each well and exposed continuously to the compounds, unless otherwise specified. 5 µl of 100 mg/ml OP50–1 were added at this stage in each well to prevent starvation. The plates were tape sealed after shaking for 2–3 m and placed at 20 °C. Every 4 days, sealers were removed to allow fresh oxygen to enter the culture and after brief shaking, plates were again resealed. The DMSO (#D8418) and tiagabine (#SML0035) that were used for post-screening assessments were purchased from Sigma Aldrich.

### Materials, equipment and system operation

Using soft lithography, we microfabricated, 3-layer and 4-layer SU-8 molds with different thicknesses to account for differences in the age-dependent body diameter, body length and head diameter between worms grown in agar plates and worms grown in liquid culture (Tables S1 and S2). Overall, the design of the device (Fig. S1) was based on our previous work^13^ with the addition of an SU-8 layer (Layer 0) that connects the side channel to the worm trap. The next layer (Layer 1) includes the worm trap and the 4-flow channels (stimulus channel, buffer channel and 2 control channels), while the thickest layer (Layer 2) includes the worm channel inlet, the flush channel and the outlet channels. PDMS was treated as previously described^13^. A Harris Uni-Core ID 0.75 mm punch was used to form the fluidic inlets for the flow channels, the worm outlet and the flush channels. A Harris Uni-Core ID 2.00 mm punch was used for the worm inlet channel. Access to the side and outlet channels was provided by polyvinyl tubes (0.023 inch I.D., 0.038 inch O.D.; BD Intramedic) connected via a steel pin (0.016 inch I.D., 0.025 inch O.D.). Access to the inlet channel was provided by a larger diameter polyethylene tubing (0.066 inch I.D, 0.095 inch O.D.; BD Intramedic) directly inserted into the port. The inlet tubing was connected to a 2-way stopcock mounted at the open bottom of a 15 ml sterile conical tube. The conical tube was connected to a valve (E12-DKBV-8FP-24D, Hyvair) that opens upon sample loading from the robotic arm (Universal XYZ Autosampler, Aurora) and to a compressed air supply that pressurizes the worm suspension in the inlet tube. The stimulus flow was delivered through a set of 4 sterile syringes (24 ml each) that contain the stimulus (glycerol) and buffer solutions (S Basal). The flow inwards the chip’s 4-flow channels was gravity-driven. The side channel was connected to a pressurized air supply and was used to unload worms from the microtrap after imaging. The chip’s outlet was connected to a vacuum controller (Multi-stage high vacuum series, GAST) to facilitate the worm’s exit from the chip. The microfluidic set up was completed with the use of three 3-way solenoid valves (LFAA1201610H, Lee Company). For all experiments, the compressed air was regulated at ~10 psi and the vacuum pressure was regulated at ~5 psi.

The imaging set up included an inverted fluorescence microscope (IX71, Olympus) equipped with a back-illuminated CCD camera (QUANTEM:512SC, Photometrics), an image splitter for FRET imaging [DV2 from MAG Biosystems equipped with a dichroic (505dcxr) and two emission filters (470/30 nm and 535/30 nm)], two optical shutters (one for fluorescence and one for white-light), a 40x oil immersion objective lens (1.3 NA, UPlanFLN, Olympus) and a z-stage system (NanoScanZ NZ400CE Nanopositioning Piezo Z Stage, prior Scientific). All hardware components were connected to a data acquisition board (BNC-2110, National Instruments) and controlled through a custom LabVIEW code.

### Computer vision and data collection

The imaging parameters used in our screen were 80 ms exposure time, a camera gain of 100, a stirring time of 70 ms, a vacuum time of 15 ms and a correlation threshold of 0.5. Once a worm was loaded, ASH neuron needed to be identified and brought into focus. This was done using a correlation-based template-matching algorithm for which a neuron template needs to be defined (Supplementary Note). The control buttons ‘Define Template’ and ‘Click when ready to define template’ enabled the user to define the template for the first worm loaded from the batch after focusing on the ASH neuron using a slider of a range of 0 to 100 µm, provided on the front panel (Fig. 1b). When the ‘Click when ready to define template’ control button is pressed, a LabVIEW GUI window pops up prompting the user to define ‘The region enclosing ASH’. This is the where the algorithm is going to search for the ASH neuron for each worm that is loaded next. Once this search region is defined, the GUI prompts the user to define the template by drawing a box around the focused ASH neuron. The user also defines the trap entry/exit region and background region which are required to determine whether the worm has been loaded or unloaded successfully. If the worm has been loaded by head then it is retained, otherwise it is flushed out. There is a provision to redefine the neuron template if the user comes across a better defined one at a later stage and while recording. If the user does not want to redefine the template, then the ‘Do Not Define Template’ control button needs to be enabled for the loaded worm. The autofocus algorithm generates a correlation score for each position of the z stage during the template-matching process and the scores are displayed as a vector on the front panel. The length of this vector is the same as the number of z stacks and can also be specified by the user in the front panel. The user can also specify a correlation threshold and if all scores in the correlation vector are below the threshold, then the ‘Neuron not found’ LED indicator lights up which means that the ASH neuron could not be brought into focus. As a result, the worm is flushed out. The correlation threshold needs to be changed by the user depending on the age of the worm as it is influenced by the fluorescence intensity of the ASH neuron.

The user can manually load the worms by using the ‘Press to manually load the worms into the vial’ and ‘Press when done manual loading’ control buttons (Fig. 1b). Each consecutive worm in the batch is loaded by pressing the ‘Load worm’ toggle control. The user can stop recording mid-batch by using the ‘Stop the Process’ control button on the front panel. There is an indicator showing the live grayscale image of each loaded worm as viewed in both CFP and YFP channels, as well as the user defined template image and the final focused image for each worm. The images are displayed on the front panel in real time and are visible at all times during auto-focusing, photobleaching and recording. Recorded imaging data from individual worms were automatically stored as a stack of images in ‘.tif’ format in a folder defined by the user.

## Electronic supplementary material

Supplementary information

Supplementary Software 1
