# Supplementary material for: An automated compound screening for anti-aging effects on the function of C. elegans sensory neurons
Source: Sci Rep. 2017 Aug 24;7:9403. doi: 10.1038/s41598-017-09651-x (PMC5570957; doi:10.1038/s41598-017-09651-x)
Supplement: Supplementary file 1 — Supplementary information [file 41598_2017_9651_MOESM1_ESM.pdf]

An automated compound screening for anti-aging effects on the function of *C. elegans* sensory neurons

Daphne Bazopoulou, Amrita R. Chaudhury, Alexandros Pantazis & Nikos Chronis

Supplementary Table S1. Thicknesses for each layer of the 3-layer microfluidic chip for worms grown in agar plates.

Worm Age (grown at 20ºC)	LAYER THICKNESS (μm)	
	Layer 1	Layer 2
Early L3*	11	53
L4†	24	42
Day 1 adult	28	45
Day 2 adult	38	50
Day 3 adult	40	55
Day 4 adult	44	65
≥Day 5 adult	48	75

Layer 0 is 7 μm thick in all cases. In Layer 1, the microfluidic worm trap has a width of 72 μm and it tapers down to a 24 μm wide opening (where the worm's nose is trapped), unless mentioned otherwise. Based on our observations, worms acquire their maximum size at Day 5 of adulthood when grown in agar plates.

* The microfluidic trap has a width of 30 μm and it tapers down to a 9 μm wide opening. In this case only, Layer 1 had 2 different thicknesses: the 4-flow channels have a thickness of 40 μm, while the microfluidic trap is 11 μm thick.

† The microfluidic trap has a width of 39 μm and it tapers down to a 12 μm wide opening.

Supplementary Table S2. Thicknesses for each layer of the 3-layer microfluidic chip for worms grown in a liquid culture.

Worm Adult Age (grown at 20ºC)	LAYER THICKNESS (µm)	
	Layer 1	Layer 2
Day 1	21	42
Day 3	38	60
Day 5	44	60
≥Day 7	47	75

Layer 0 was 7 µm thick. In Layer 1, the microfluidic worm trap has a width of 72 µm and it tapers down to a 17 µm wide opening (where the worm's nose is trapped), unless mentioned otherwise. Based on our observations, worms acquire their maximum size at Day 7 of adulthood when grown in a liquid culture.

Supplementary Table S3. Time needed to load and unload single worms from the chip.

Worm Adult Age (grown at 20°C)	Loading (s)	Unloading (s)
Day 1	3	3
Day 3	4	4
Day 5	4	4
≥Day 7	5	5

A constant vacuum of 5 psi was applied to the outlet of the microfluidic chip. Compressed air of 10 psi was applied at the inlet of the microfluidic trap and the side channel during worm loading/unloading.

Supplementary Table S4. List of the names of compounds along with the SMILES representation of their chemical structures and their respective target category.

Target Category	*Name*	*Molecular Structure*
Serotonin	Nefazodone	Cl.CCc1nn(CCCN2CCN(CC2)c2cccc(Cl)c2)c(=O)n1CCOc1ccccc1
	Olanzapine	CN1CCN(CC1)C1=Nc2ccccc2Nc2sc(C)cc12
	Sumatriptan succinate	OC(=O)CCC(O)=O.CNS(=O)(=O)Cc1ccc2[nH]cc(CCN(C)C)c2c1
	Venlafaxine	Cl.COc1ccc(cc1)C(CN(C)C)C1(O)CCCCC1
	Altanserin	O.Cl.Fc1ccc(cc1)C(=O)C 1CCN(CCn2c(=S)[nH]c3ccccc3c2=O)CC1
	Duloxetine	Cl.CNCC[C@H](Oc1cccc2ccccc12
	Fluperlapine	CN1CCN(CC1)C1=Nc2cc(F)ccc2Cc2ccccc12
	RU 24969	OC(=O)CCC(O)=O.COc1ccc2[nH]cc(C3=CCNCC3)c2c1
	Zolmitriptan	CN(C)CCc1c[nH]c2ccc(C[C@H]3COC(=O)N3)cc12
	Tramadol	Cl.COc1cccc(c1) [C@@]1(O)CCCC[C@@H]1CN(C)C
	Dolasetron	CS(O)(=O)=O.O=C(O[C@H]1C[C@H]2C[C@H]3C[C@@H](C1)N2CC3=O)c1c[nH]c2ccccc12
	L-694,247	O.CS(=O)(=O)Nc1ccc(Cc2noc(n2)-c2ccc3[nH]cc(CCN)c3c2)cc1
	CGS 12066B	OC(=O)\C=C/C(O)=O.CN1CCN(CC1)c1nc2cc(ccc2n2cccc12)C(F)(F)F
	Mirtazapine	CN1CCN2C(C1)c1ccccc1Cc1cccnc21
	N-(3-Trifluoromethylphenyl) piperazine hydrochloride	Cl.FC(F)(F)c1cccc(c1)N1CCNCC1
	(3-Trifluoromethylphenyl) piperazine hydrochloride	Cl.FC(F)(F)c1cccc(c1)N1CCNCC1
	Pirenperone	Cc1nc2ccccn2c(=O)c1CCN1CCC(CC1)C(=O)c1ccc(F)cc1
	Zacopride	O.Cl.COc1cc(N)c(Cl)cc1C (=O)NC1CN2CCC1CC2
	Trazodone hydrochloride	Cl.Clc1cccc(c1)N1CCN(CCCn2nc3ccccn3c2=O)CC1
	Escitalopram oxalate	OC(=O)C(O)=O.CN(C)CCC[C@]1(OCc2cc(ccc12)C#N)c1ccc(F)cc1
	SR 57,227A	Cl.NC1CCN(CC1)c1cccc(Cl)n1
	Pizotyline	OC(=O)\C=C/C(O)=O.CN1CCC(CC1)=C1c2ccsc2CCc2ccccc12
	Perospirone	Cl.O=C1[C@H]2CCCC[C@H]2C(=O)N1CCCCN1CCN(CC1)c1nsc2ccccc12
Dopamine	Risperidone	Cc1nc2CCCCn2c(=O)c1CCN1CCC(CC1)c1noc2cc(F)ccc12
	Lobeline	Cl.CN1[C@H](C[C@H](O)c2ccccc2)CCC[C@@H]1CC(=O)c1ccccc1
	Haloperidol	Cl.OC1(CCN(CCCC(=O)c2ccc(F)cc2)CC1)c1ccc(Cl)cc1
	Pergolide mesylate	CS(O)(=O)=O.[H][C@@]12Cc3c[nH]c4cccc(c34)[C@@]1([H])C[C@@H](CSC)CN2CCC
	Rimcazole	O.Cl.C[C@H]1CN(CCCn2c3ccccc3c3ccccc23)C[C@@H](C)N1
	Phenothiazine	N1c2ccccc2Sc2ccccc12
	SB 205607	O.Br.CN1CC[C@@]2(Cc3nc4ccccc4cc3C[C@H]2C1)c1cccc(O)c1
	Raclopride	CCN1CCC[C@H]1CNC(=O)c1c(O)c(Cl)cc(Cl)c1OC
	Disulfiram	CCN(CC)C(=S)SSC(=S)N(CC)CC
	Modafinil	NC(=O)CS(=O)C(c1ccccc1)c1ccccc1
	SKF 83566	Br.CN1CCc2cc(Br)c(O)cc2C(C1)c1ccccc1
	Trifluoperazine dihydrochloride	Cl.CN1CCN(CCCN2c3ccccc3Sc3ccc(cc23)C(F)(F)F)CC1
	Pemoline	NC1=NC(=O)C(O1)c1ccccc1
	Levosulpiride	CCN1CCC[C@H]1CNC(=O)c1cc(ccc1OC)S(N)(=O)=O
	Pramipexole	CCCN[C@H]1CCc2nc(N)sc 2C1
	Amisulpride	CCN1CCCC1CNC(=O)c1cc(c(N)cc1OC)S(=O)(=O)CC
	Amfebutamone	CC(NC(C)(C)C)C(=O)c1cccc(Cl)c1
GABA	Flumazenil	CCOC(=O)c1ncn- 2c1CN(C)C(=O)c1cc(F)ccc-21
	Diazepam	CN1c2ccc(Cl)cc2C(=NCC1=O)c1ccccc1
	Nitrazepam	[O-][N+](=O)c1ccc2NC(=O)CN=C(c3ccccc3)c2c1
	Noreleagnine	C1Cc2c(CN1)[nH]c1ccccc21

	Stiripentol	CC(C)(C)C(O)\C=C\c1ccc2OCOc2c1
	Zaleplon	CCN(C(C)=O)c1cccc(c1)-c1ccnc2c(cnn12)C#N
	Lorazepam	OC1N=C(c2ccccc2Cl)c2cc(Cl)ccc2NC1=O
	Clobenpropit	Clc1ccc(CNC(=N)SCCCc2c[nH]cn2)cc1
	Picrotoxinin	[H][C@@]12C[C@@]3(O)[C@@H]4[C@@H]([C@@H](OC4=O)[C@H]4OC(=O)[C@]1(O2)[C@@]34C)C(C)=C.CC(C)(O)[C@@H]1[C@H]2OC(=O)[C@@H]1[C@]1(O)C[C@H]3O[C@]33C(=O)O[C@H]2[C@]13C
	Alprazolam	Cc1nnc2CN=C(c3ccccc3)c3cc(Cl)ccc3-n12
	Tiagabine	Cl.Cc1ccsc1C(=CCCN1CCC[C@H](C1)C(O)=O)c1sccc1C
	Honokiol	Oc1ccc(cc1CC=C)-c1cc(CC=C)ccc1O
Acetylocholine	Benactyzine	Cl.CCN(CC)CCOC(=O)C(O)(c1ccccc1)c1ccccc1
	Donepezil	Cl.COc1cc2CC(CC3CCN(Cc4ccccc4)CC3)C(=O)c2cc1OC
	Galanthamine hydrobromide	Br.COc1ccc2CN(C)CC[C@@]34C=C[C@H](O)C[C@@H]3Oc1c24
	Vesamicol hydrochloride	Cl.O[C@@H]1CCCC[C@H]1N1CCC(CC1)c1ccccc1
	Pancuronium	[Br].CC(=O)OC1C(C[C@H]2C3CC[C@H]4C[C@H](OC(C)=O)C(CC4(C)[C@H]3CCC12C)[+]1(C)CCCCC1) [N+]1(C)CCCCC1
	Pilocarpine hydrochloride	Cl.CC[C@H]1[C@@H](Cc2cncn2C)COC1=O
	Bifemelane	Cl.CNCCCCOc1ccccc1Cc1ccccc1
	Nornicotine	C1CNC(C1)c1cccnc1
	Cotinine	CN1[C@@H](CCC1=O)c1cccnc1
Epi/Nor-epinephrine	Lofexidine	Cl.CC(Oc1c(Cl)cccc1Cl)C1=NCCN1
	Maprotiline hydrochloride	Cl.CNCCCC12CCC(c3ccccc13)c1ccccc21
	Prazosin	O.Cl.COc1cc2nc(nc(N)c2cc 1OC)N1CCN(CC1)C(=O)c1ccco1
	Piribedil	O.Cl.C(N1CCN(CC1)c1ncccn1)c1ccc2OCOc2c1
	Atomexetine	Cl.CNCC[C@@H](Oc1ccccc1C)c1ccccc1
Glutamate	Dextrorphan D-Tartrate	OC(C(O)C(O)=O)C(O)=O.CN1CC[C@@]23CCCC[C@@H]2[C@@H]1Cc1ccc(O)cc31
	Ifenprodil	O.OC(C(O)C(O)=O)C(O)=O.CC(C(O)c1ccc(O)cc1)N1CCC(Cc2ccccc2)CC1
	Memantine	Cl.C[C@@]12C[C@H]3C[C@@](C)(C1)C[C@](N)(C3)C2
Na^{+} channels	Oxcarbazepine	NC(=O)N1c2ccccc2CC(=O)c2ccccc12
	Lamotrigine	Nc1nnc(c(N)n1)-c1cccc(Cl)c1Cl
	Riluzole	Cl.Nc1nc2ccc(OC(F)(F)F)cc2s1
	Ropivacaine	Cl.CCCN1CCCC[C@H]1C(=O)Nc1c(C)cccc1C
	Lidocaine	CCN(CC)CC(=O)Nc1c(C)cccc1C
Ca^{+2} channels	Lomerizine	Cl.COc1ccc(CN2CCN(CC2)C(c2ccc(F)cc2)c2ccc(F)cc2)c(OC)c1OC
	Diazoxide	CC1=NS(=O)(=O)c2cc(Cl)ccc2N1
Antioxidant	Ebselen	O=c1n([se]c2ccccc12)-c1ccccc1
	7- Nitroindazole	[O-][N+](=O)c1cccc2c[nH]nc12
	Epigallocatechin-3-monogallate	Oc1cc(O)c2C[C@@H](OC(=O)c3cc(O)c(O)c(O)c3) [C@H](Oc2c1)c1cc(O)c(O)c(O)c1
Opiod receptor	GR 89696	OC(=O)\C=C\C(O)=O.COC(=O)N1CCN(C(CN2CCCC2)C1)C(=O)Cc1ccc(Cl)c(Cl)c1
	Nalbuphine	Cl.OC1CC[C@@]2(O)[C@H]3Cc4ccc(O)c5OC1[C@]2(CCN3CC1CCC1)c45
	Naltrindole	O.Cl.Oc1ccc2CC3N(CC4CC4)CC[C@@]45[C@@H](Oc1c24)c1[nH]c2ccccc2c1C[C@@]35O
Adenosine receptor	CGS 15943	Nc1nc2ccc(Cl)cc2c2nc(nn12)-c1ccco1
Cannabinoid receptor	AM-251	Cc1c(nn(c1-c1ccc(I)cc1)-c1ccc(Cl)cc1Cl)C(=O)NN1CCCCC1
PDE inhibitor	Rolipram	COc1ccc(cc1OC1CCCC1)C1CNC(=O)C1
	Icariin	COc1ccc(cc1)c1oc2c(CC=C(C)C)c(O[C@@H]3O[C@H](CO)[C@@H](O)[C@H](O)[C@H]3)cc(O)c2c(=O)c1O[C@@H]1O[C@@H](C)[C@H] (O)[C@@H](O)[C@H]1O

Combinatorial/ Other function	Moxonidine	Cl.COc1nc(C)nc(Cl)c1NC1=NCCN1
	Hyperoside	OC[C@H]1O[C@@H](Oc2c(oc3cc(O)cc(O)c3c2=O)c2ccc(O)c(O)c2)[C@H](O)[C@@H](O)[C@H]1O
	Isoquercetrin	OC[C@H]1O[C@@H](Oc2c(oc3cc(O)cc(O)c3c2=O)c2ccc(O)c(O)c2)[C@H](O)[C@@H](O)[C@@H]1O
	Pterostilbene	COc1cc(OC)cc(c1)\C=C\c1ccc(O)cc1
	Felbamate	O.NC(=O)OCC(COC(N)=O)c1ccccc1
	Calcitriol	C[C@H](CCCC(C)(C)O)[C@H]1CC[C@@H]2[C@]1(C)CCC\C2=C/C=C1/C[C@@H](O)C[C@H](O)C1=C
	Physostigmine	OS(O) (=O)=O.CNC(=O)Oc1ccc2N(C)C3N(C)CC[C@@]3(C)c2c1
	Ticlopidine	Cl.Clc1ccccc1CN1CCc2sccc2C1
	1-Benzylimidazole	C(c1ccccc1)n1ccnc1
	Prostaglandin	CCCCC[C@H] (O)\C=C\[C@H]1[C@H](O)CC(=O)[C@@H]1CCCCCCC(O)=O
	Sibutramine	CC(C)CC(N(C)C)C1(CCC1)c1ccc(Cl)cc1
	Nicotinamide	NC(=O)c1cccnc1
	Loxapine succinate	OC(=O)CCC(O)=O.CN1CCN(CC1)C1=Nc2ccccc2Oc2ccc(Cl)cc12
	Famciclovir	CC(=O)OCC(CCn1cnc2cnc(N)nc12)COC(C)=O
	Doxepin hydrochloride	Cl.CN(C)CC\C=C1\c2ccccc2COc2ccccc12
	Nobiletin	COc1ccc(cc1OC)-c1cc(=O)c2c(OC)c(OC)c(OC)c(OC)c2o1
	Phenelzine sulfate	OS(O) (=O)=O.NNCCc1ccccc1
	Mesoridazine	OS(=O)(=O)c1ccccc1.CN1CCCCC1CCN1c2ccccc2Sc2ccc(cc12)S(C)=O
	Indatraline	Cl.CN[C@@H]1C[C@H](c2ccccc12)c1ccc(Cl)c(Cl)c1
	Idebenone	COC1=C(OC)C(=O)C(CCCCCCCCCCO)=C(C)C1=O
	Taxifolin	O[C@H]1[C@H](Oc2cc(O)cc(O)c2C1=O)c1ccc(O)c(O)c1
	Piceid	OC[C@H]1O[C@@H](Oc2cc(O)cc(\C=C\c3ccc(O)cc3)c2)[C@H](O)[C@@H](O)[C@@H]1O

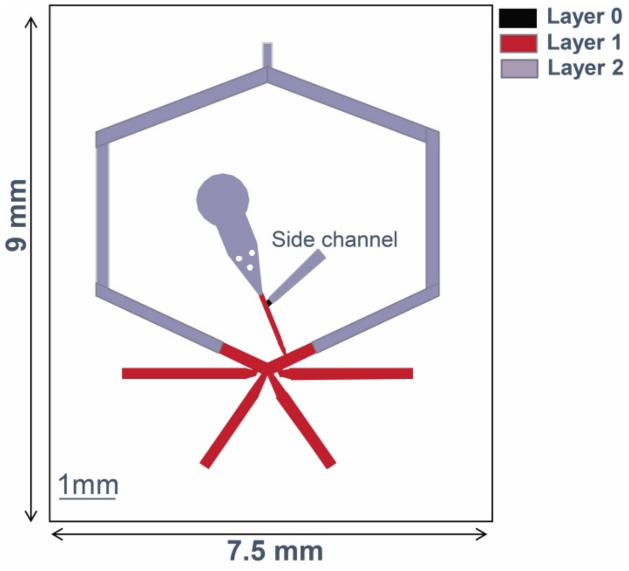

Supplementary Figure S1. Schematic of the 3-layer microfluidic chip (top view). The side channel is used to flush out the worm from the microfluidic trap. Layer 0 is used to connect the side channel to the microfluidic trap.

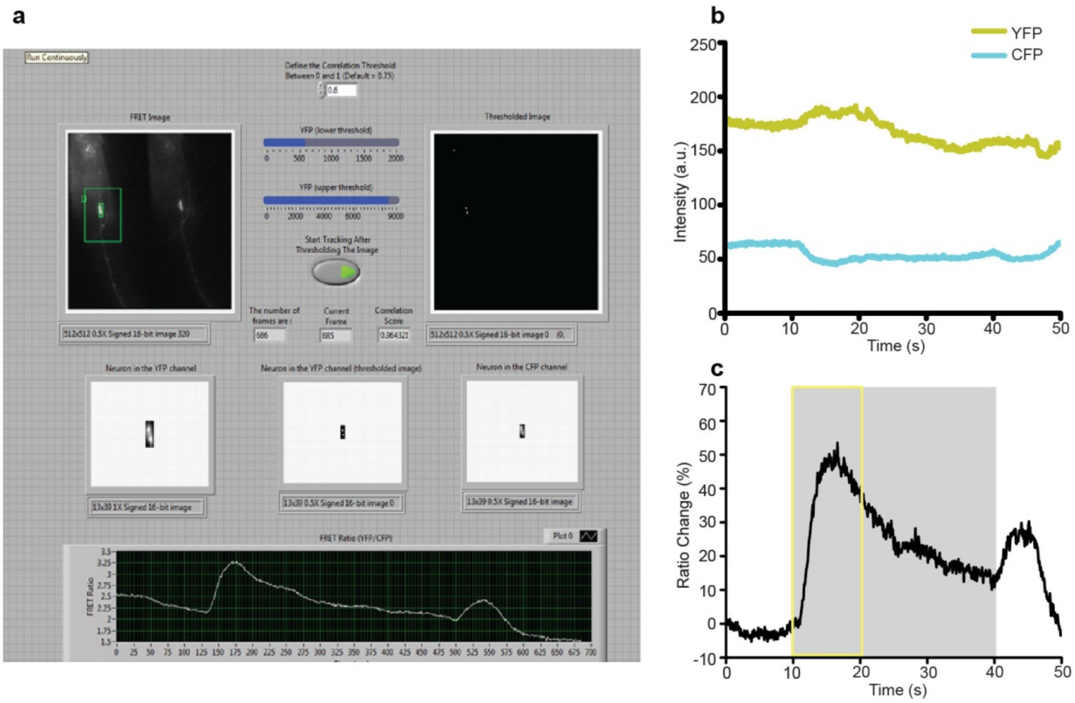

Supplementary Figure S2. a) Snapshot of the Labview panel built for extracting the YFP/CFP ratio from the recorded unprocessed YFP and CFP channel data (Fig. 1b). The program allows the user to define the background intensity and add a threshold for the neuronal soma for automated intensity tracking b) Recorded unprocessed intensity signal from the YFP and CFP channels (yellow and cyan lines respectively) during a 30 s application of 1 M glycerol. c) The YFP/CFP FRET ratio over time as a percentage change from the baseline and corrected for photobleaching. The shaded grey box indicates the presence of the stimulus. The yellow box indicates the time period for which the FRET signal was quantified.

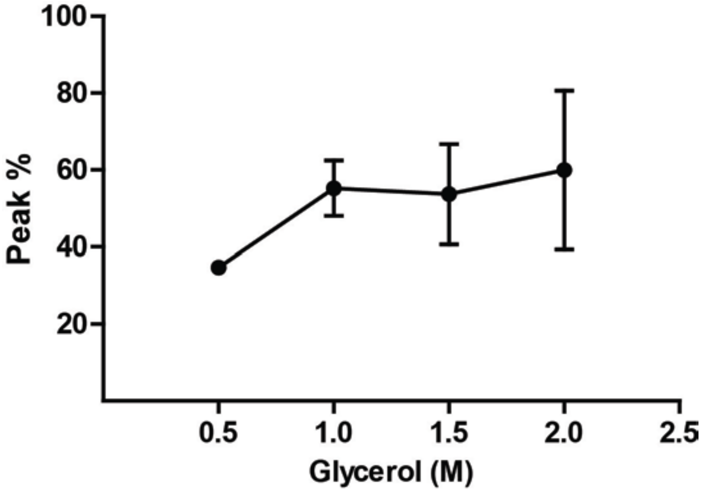

Supplementary Figure S3. a) Averaged peak of ASH responses at 0.5 M, 1 M, 1.5 M and 2M of glycerol. For concentrations > 1 M there is no further increase in the peak of calcium transients. Error bars indicate SEM. Between 18 and 22 worms were imaged for each concentration in duplicate. No statistical difference for concentrations > 1M compared to 1M glycerol, one-way ANOVA followed by *t*-test pairwise analysis.

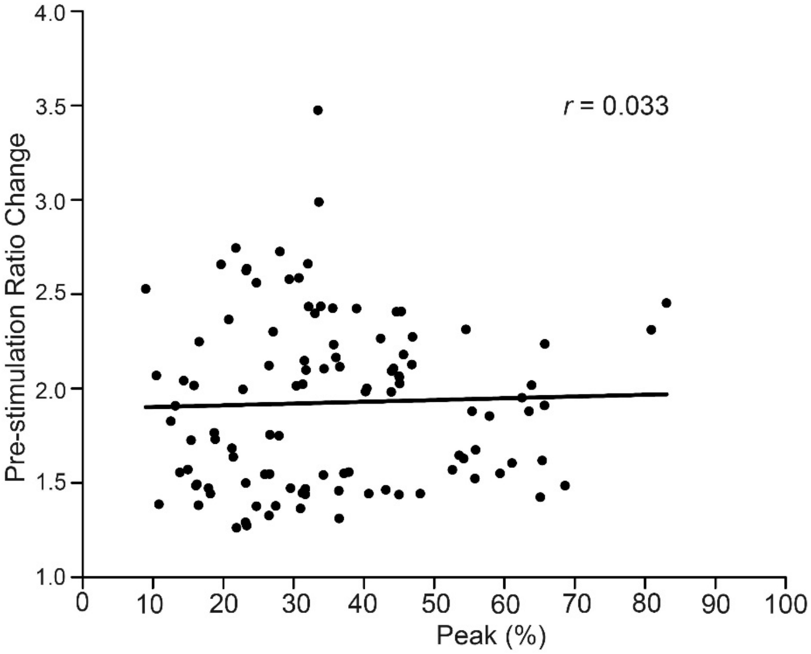

Supplementary Figure S4. Correlation between pre-stimulation ratio change and peak response (%) of ASH neuron for Day 5 adults. 1 M glycerol was used as stimulus. A total of 108 Day 5 adults were imaged in triplicate. *r* = Pearson's correlation coefficient.

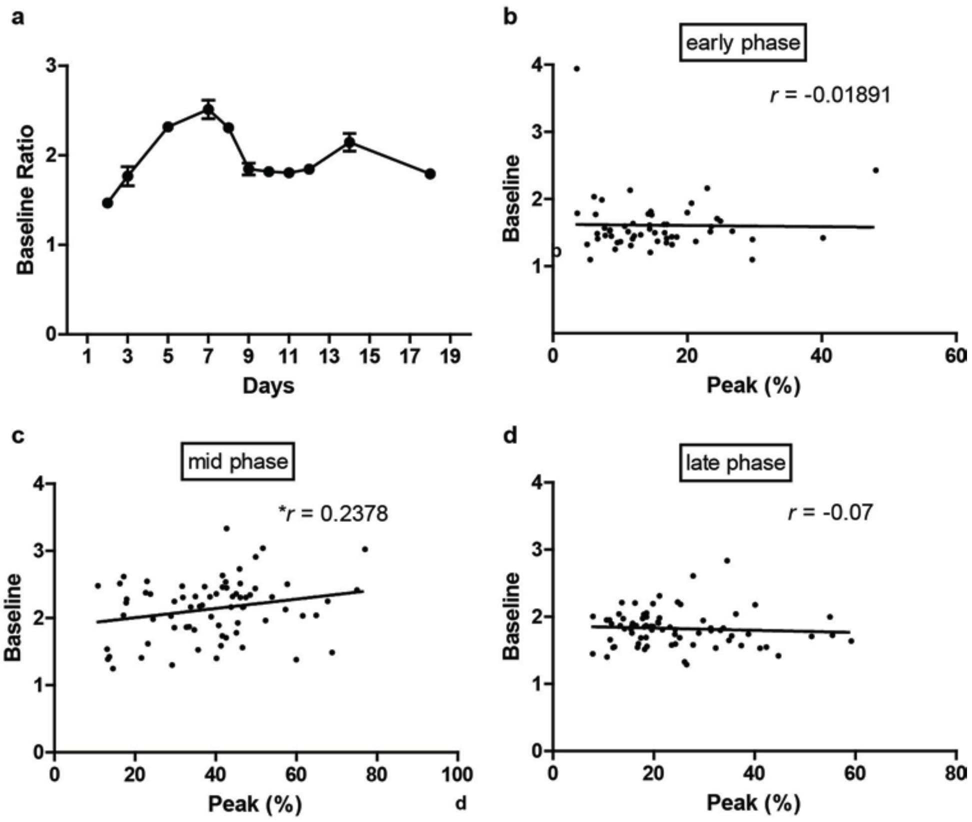

Supplementary Figure S5. a) Baseline ratio of ASH responses during adulthood. b), c) and d) depict correlation between baseline ratio and peak responses (%) in early (c), mid (d) and late (e) phase of observations. 1 M glycerol was used as stimulus. A total of 55, 71 and 75 worms are shown in b), c) and d) respectively. *r* = Pearson's correlation coefficient. **p*<0.5.

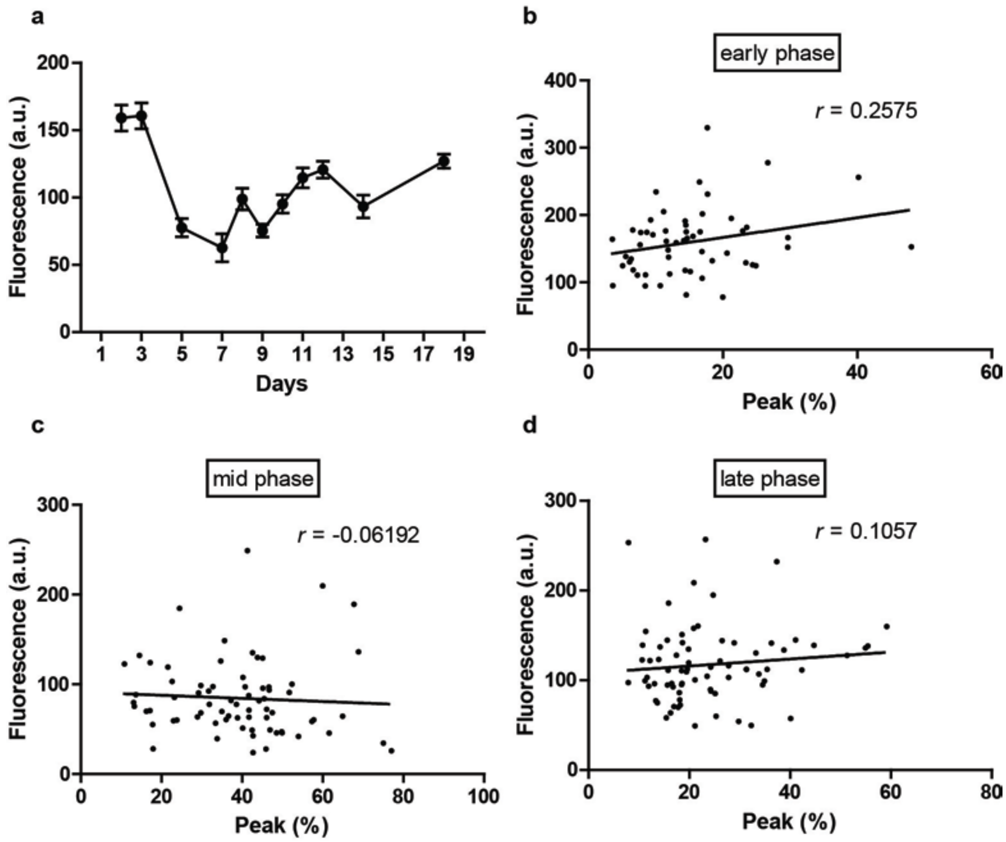

Supplementary Figure S6. a) Fluorescence intensity of TNXL sensor over different time points during adulthood. b), c) and d) depict correlation between fluorescence intensity and peak responses (%) in early (c), mid (d) and late (e) phase of observations. 1 M glycerol was used as stimulus. In a) between 39 and 58 worms were imaged for each time point in triplicate. A total of 55, 71 and 75 worms are shown in b), c) and d) respectively. *r* = Pearson's correlation coefficient.

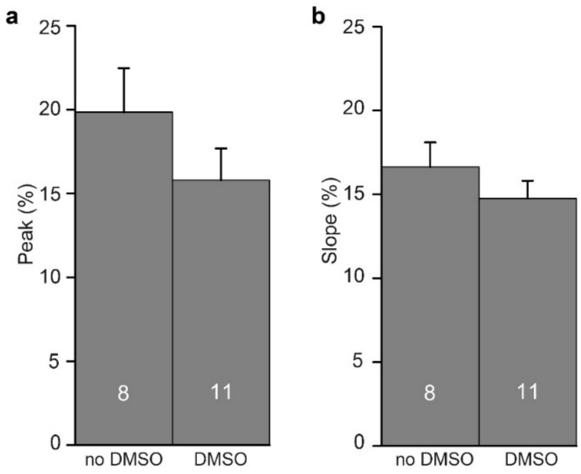

Supplementary Figure S7. Effect of 0.4% DMSO ASH responses of Day 12 adults. Bar graphs depict the averaged peak (a) and slope (b) of ASH responses in the presence or absence of 0.4% DMSO. 1 M glycerol was used as stimulus. Error bars indicate SEM. Numbers on bars indicate number of worms imaged. No statistical difference, two-tailed *t*-test.

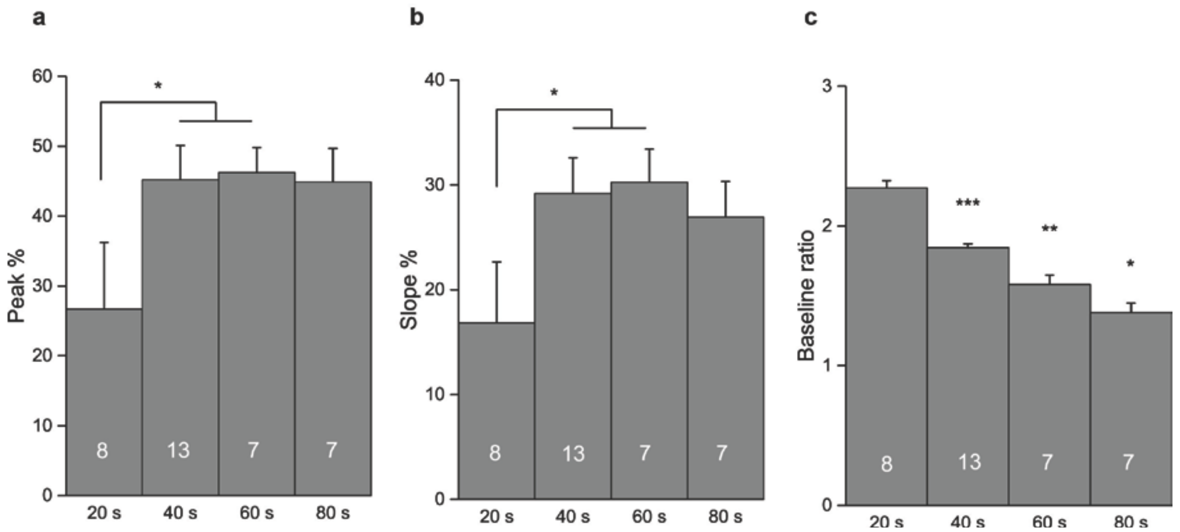

Supplemental Figure S8. ASH ratiometric transients in response to 1 M glycerol obtained for different pre-stimulation exposure times. Bar graphs depict averaged peak (a) and slope (b) of ASH responses after each indicated exposure time. In (c) the averaged baseline ratio for each exposure time is shown. Numbers on bars indicate number of worms imaged. *$p < 0.05$, **p<0.01, ***p< 0.001, one-way ANOVA followed by Bonferroni's test for multiple comparisons.

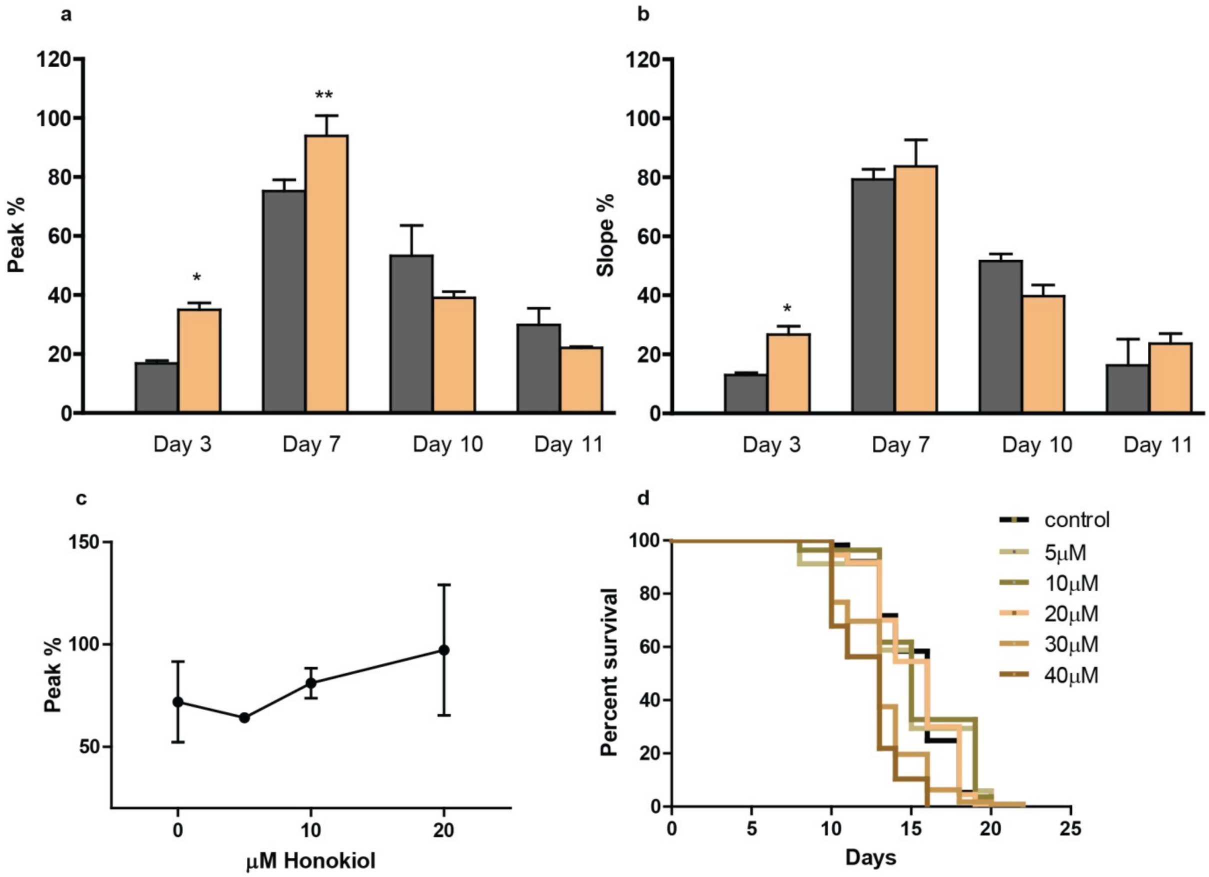

Supplemental Figure S9. Bar graphs depict the averaged peak (a) and slope (b) of ASH responses during adulthood after administrating of 20 μM honokiol in Day 1 adults. (c) Dose response curve to 5, 10 and 20 μM of honokiol. (d) Lifespan assay at lindicated concentrations of honokiol. Error bars indicate SEM. For a) and b) and c) between 21 and 35 worms were imaged. Experiments were carried out in triplicate. **p*<0.5, ***p*<0.01, two-way ANOVA followed by Bonferroni's test for multiple comparisons. For lifespan assay, median survival: control =16, (n=113); 5 μM honokiol = 15, (n=34); 10 μM honokiol = 15, (n=55); 20 μM honokiol = 16, (n=110); 30 μM honokiol =13, (n=112), *p* <0.5 compared to control; 40 μM honokiol =13, (n=87), *p* <0.5 compared to control. Statistical analysis: log-rank (Mantel-Cox) test.

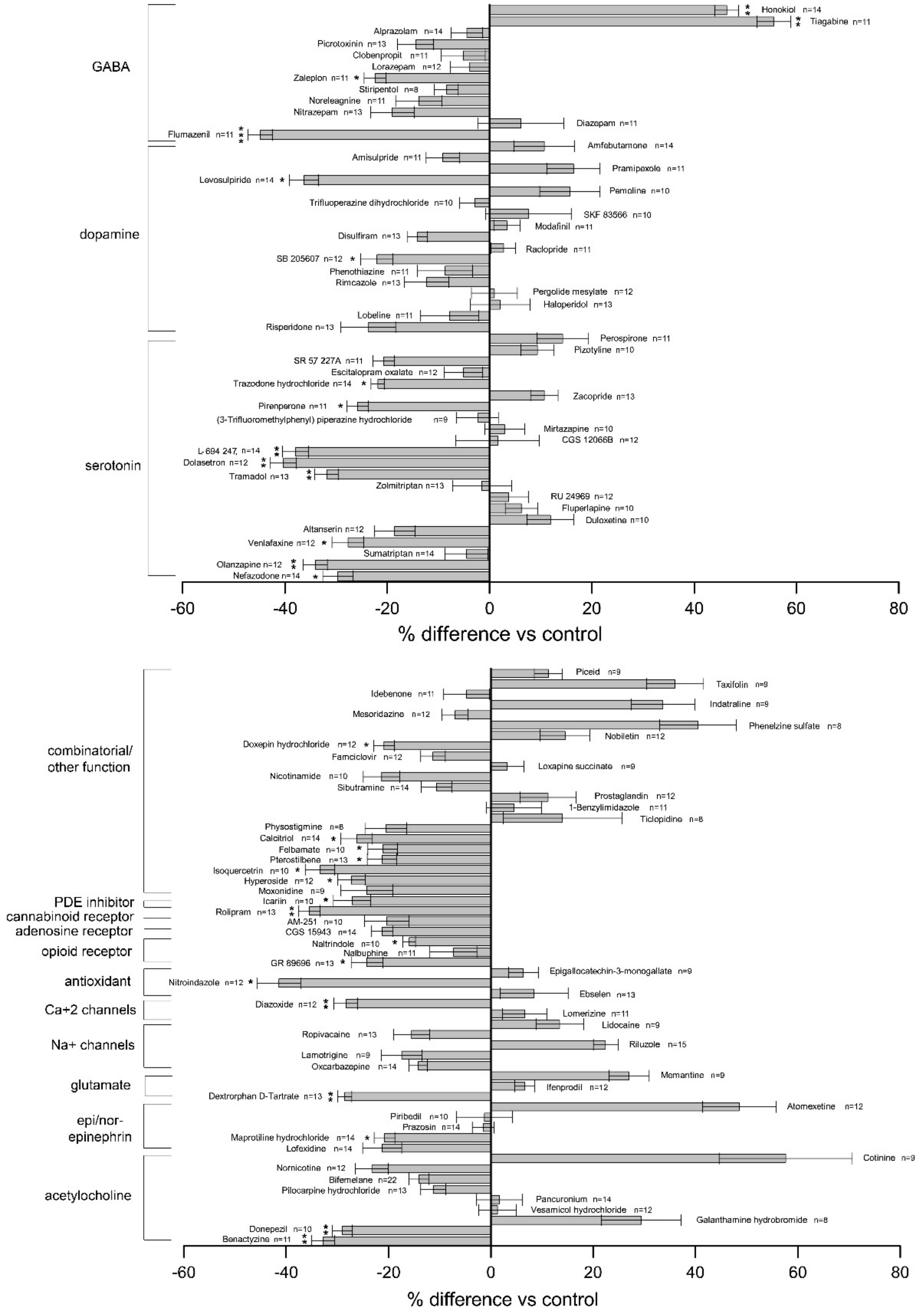

Supplementary Figure S10. Evaluation of calcium transient data from the pilot screen. Worms were incubated at 20 ^{0}C in the presence of the chemical library starting at Day 5 of adulthood. Peak of the ASH

response was measured at Day 12 in response to 1 M glycerol and the percent differences were calculated. Bars represent the total mean values from all experiments. Error bars denote SEM and n indicates the total number of worms imaged in each case. $*p < 0.05$, $**p < 0.01$, one-way ANOVA followed by *t*-test pairwise analysis to compare each compound treatment to control.

Supplementary Note

Autofocus Algorithm. Depending on ASH soma shape and fluorescent intensity, and for autofocus analysis purposes we classified Day 1 to Day 4 adults as ‘young worms’ and Day 5 to Day 12 worms as ‘old worms’. The ASH neuron is visibly much brighter in the young worms compared to the old ones. In cases of old worms, the background can be as bright as the ASH neuron. Also, young worms are more mobile in the traps so the position of the neuron changes frequently as the z stage moves. This frequent shift in position leads to a small but perceptible change in the shape and appearance of the ASH neuron. The older worms are much thicker and therefore sit more securely in the worm traps. Thus, the position of the ASH neuron remains fairly constant and the shape is retained. For each worm in the batch, the z stage performs a vertical scan (maximum range of 100 μm) and acquires a number of frames corresponding to sequential increments from the reference position on the z axis, as specified by the user at the start of each experiment. These numbers may be modified in the middle of the batch but cannot be altered in the middle of the recording. The objective of the autofocus algorithm is to choose the best focus frame from the total number of frames acquired after a new worm is loaded into the microfluidic trap.

The primary method employed in the autofocus algorithm is template-based object recognition applied to grayscale images. A convolution or template mask is used which is tailored to a specific feature of the search image, the ASH neuron. The convolution output will be highest at places where the image structure matches the mask structure and where large image values get multiplied by large mask values. A part of the search

image is picked as the template. The z stage is moved using a slider on the front panel of the LabVIEW application and the ASH neuron of the first worm that is loaded by head is brought into focus. The position of the focal plane (corresponding z stage position) is recorded as the ‘approximate focal plane’. The search region in the image is then defined by the user. The search area is not comprised of the whole image as there are other bright objects which usually appear at the periphery. Although these do not correspond to the neuron of interest, they can appear similar to its shape and/or intensity and be falsely processed by the algorithm. This could sometimes pose a challenge especially in older worms. A smaller search area leads to a faster matching time as template-matching is a time intensive process. After defining the search image, the focused ASH neuron is selected as the template or the filter mask (a small box enclosing the ASH neuron is drawn using the cross hair in the LabVIEW GUI). The center of the template is then moved over each point (pixel location) of the search region and a correlation score is generated for each point over this entire area. Correlation is a measure of the degree to which two variables agree, not necessarily in actual value but in general behavior. The variables in this case are the pixel values of the template and the search image. As all possible positions of the template with respect to the search image are considered, the position with the highest correlation score is ultimately the best focused position. The following correlation formula is implemented in MATLAB to calculate the correlation score for each pixel location in the search image.

$$cor = \frac{\sum_{i=0}^{N-1} (x_i - \overline{x}) \cdot (y_i - \overline{y})}{\sqrt{\sum_{i=0}^{N-1} (x_i - \overline{x})^2 \cdot \sum_{i=0}^{N-1} (y_i - \overline{y})^2}}$$

x is the template gray level image

x is the average grey level in the template image

y is the source image section

y is the average grey level in the source image

N is the number of pixels in the section image

(N= template image size = columns * rows)

The scores are normalized to fall within a range of 0 to 1 with values below 0.5 representing low correlation and values above 0.5 representing moderate to high correlation. The highest correlation score form each frame is stored in a vector and displayed in the front panel as the correlation matrix. Therefore, for a given worm, the number of stacks equals the number of elements in the correlation score matrix. The z position corresponding to the maximum correlation score is termed as the ‘actual focal plane’. For the first worm in the batch, the approximate focal plane is the same as the actual focal plane as this worm is used to define the template ASH neuron. The highest correlation score is also found to be about 0.995 indicating a nearly perfect correlation. For the next worms, the highest correlation score is lower than 0.9 as the neurons do not exactly match the template. If the highest correlation score is below the threshold specified by the user, the worm is flushed out. The correlation threshold is however adjustable by the user in the middle of a batch. Also, positioning the slider at the approximate focal plane at the start of the experiment expedites ASH focusing for subsequent worms within ten steps of z stage movement. This is based on our observation that the ‘actual focal plane’ for worms of roughly the same age correspond in position with each other. Rotation and scaling affects template-matching adversely

leading to inexact matches and low correlation scores. For old worms, there is not much variability in the appearance of the neuron so template-matching gives relatively high correlation scores (0.7-0.8). For younger worms however, there is a fair amount of variability in both position and shape of the ASH neuron. Thereby, the resulting correlation scores generated by the template matching algorithm are lower. For this purpose, an edge detection code using the Sobel method was written in MATLAB and applied to all the acquired frames for a given worm. The MATLAB function ‘edge’ takes each grayscale image (search image for each of the frames) as its input, and returns a binary image of the same size, with 1's where the function finds edges and 0's at all other positions. The Sobel function automatically determines the sensitivity threshold so it ignores all edges which are below the threshold. As the ASH neuron is the brightest object in the field of view, especially in young worms, the edges of the neuron are always selected. It was noted that the frame with the highest Sobel threshold value was closest to the actual focal plane (best focus frame). The edge detection method works better than template-matching based on highest correlation scores for young worms, whereas the template-matching method is more suitable for old worms. The user can select either of the two methods using a toggle control on the front panel.

In a newest version of the LabVIEW automation code, the autofocus algorithm combines both the edge detection and the template-matching methods to determine best focus plane which works both for old and young worms. For each worm, the frames with the ten highest correlation scores are extracted after the z scan is run. The Sobel edge detection operator is applied to these ten frames and the one with the highest Sobel threshold is then selected as the best focus frame. This algorithm seemed to work fairly

well with both young and old worms after preliminary experiments but still needs to be tested on worms of varying age groups.

LabVIEW automation code block diagram. The main automation LabVIEW VI has 8 stacked frames contained in an indexed master while loop. The control for this outermost while loop can be enabled by the user to stop the batch process. Otherwise, the contents of the loop are repeated as many times as the number of worms in a batch, with the index of the loop keeping track of this number.

Frame 1 of the main loop. This frame contains a case structure and the value of the while loop index corresponds to the first iteration of the loop and therefore the first worm in the batch is defined as the true condition for the case structure. The camera is controlled by the Scientific Imaging ToolKit for LabVIEW. The subVI exopenglobalcam.vi must be opened and run before the main program. The main VI uses a global camera handle. For the true Boolean value, the program checks if the global camera handle is open. If yes, the program continues and uses the handle. If not, the program generates an error code and stops. Consequently, the subVI excloseglobalcam.vi has to be run before leaving LabVIEW. This sequence also initializes the external control devices, namely the Photometrics camera and the PCI-6014 card to control the shutter and the valves. The VISA resources for the robotic arm, shutter and z stage are hardwired to the VISA configure serial ports in LabVIEW. The true case contains a stacked sequence structure which turns off the valves in the system and initializes the digital and analog outputs. The valves responsible for worm loading, unloading and stimulus delivery are interfaced to the computer via an NI USB 6009 8 input 14 bit multifunctional I/O DAQ

board and are controlled by the LabVIEW NI DAQmx Express Vis (NI Measurement and Automation Explorer). Once the smart shutter is initialized, the white light shutter is opened. The z stage is controlled by a RS 232 serial connection via a USB interface. The user moves the slider and controls the vertical movement of the z stage. Before the first worm is loaded into the trap, the user has to focus in the live trap image and then press the 'Click proceed after focusing the image' control button to continue. When the user chooses to proceed, LabVIEW prompts the user to define a rectangular region enclosing the ASH neuron, or the search region with a cross hair cursor. The coordinates of the search region are registered and for every worm which is subsequently loaded in the batch and the algorithm (Supplementary Note 1) searches for the ASH neuron in the same area of the image. LabVIEW also prompts the user to define a background area. The IMAQ light meter rectangle is used to find the mean background intensity.

Frame 2 of the main loop. This frame introduces a 60 s delay (wait VI) before the next worm is loaded.

Frame 3 of the main loop. An additional stacked frame sequence which allows manual loading of worms is introduced. The first frame in the stacked sequence has a while loop. In the loop, if the 'Press to manually load worms into the vial' is activated, the inlet valve is opened. When the 'Press when done manual loading' is enabled, the inlet valve closes followed by a 3ms delay. There is also a case structure, where the 'true' condition corresponds to the first worm that is loaded. In this case, LabVIEW prompts the user to select the 'Worm entrance area' followed by the 'Region near the end of the trap'. The standard deviation of the pixel intensities in the background region, worm entrance region and the region near the end of the trap as defined by the user is calculated using

the IMAQ Light Meter (Rectangle) VI. The standard deviation of the background intensity is subtracted from the standard deviation of the entrance region and the region near the end of the trap. If the difference is greater than the ‘Threshold cut off to recognize the worm’, specified by the user, and the ‘Manual Control Pressure Inlet’ is disabled, then the pressure is released at the inlet trap and the worm is flushed out. Otherwise, the worm is positioned at the end of the trap by the inlet valves. A count is run from 0 to 50 and if the worm is still in the trap, then the inlet pressure is applied for 250 ms and then released. Otherwise, the inlet pressure continues to be applied and the worm is flushed out. Loading process for each worm is activated once the wait time, specified by the user using the control ‘Enter time to wait before new sample load, runs out. The while loop is terminated after the worm is loaded in the trap. The second frame in the stacked sequence contains subVIs, Robotic Arm Control (SubVI) and NIQmx DAQ express VI, for controlling the robotic arm. The latter VI also alerts the user in the case the robotic arm is not functioning properly e.g. due to bubbles in the tubing system. The user defined controls for the robotic arm are the stirring and the vacuum time.

Frame 4 of the main loop: For the first worm, the user has to bring the ASH neuron in focus by adjusting the slider on the front panel. The ASH neuron for the first worm serves as the template image for the autofocus algorithm. This frame has a case structure placed in a while loop. The case structure is further divided into stacked sequences. Before adjusting the slider to focus the neuron, the user must enable the control ‘Define Template’ which is the true condition for the outer case structure. When ‘Define Template’ is enabled, CFP fluorescence is turned on and the white light shutter is closed. The slider controls the vertical movement of the microscope z stage via a RS232 serial

connection and LabVIEW serial read and write VISA drivers. Before moving the z stage, the serial port is configured (baud rate, data bits, parity, stop bits and flow control). The best focus z stage position is displayed as the 'Initial focus z position'. The instructions for controlling the z stage with the slider are in a while loop. When the user enables the control 'Click when ready to define the template', the while loop is terminated and control moves on the next stacked sequence in the case structure where the 'Initial focus z position' is read from the z stage controller. In the next frame of the inner stacked sequence, the CFP fluorescence is turned off. LabVIEW prompts the user to define a template where the user needs to box the focused ASH neuron using a cross hair cursor. The LabVIEW subVIs used in this case are the IMAQ Select Rectangle, IMAQ Extract, IMAQ Array to Image and IMAQ Image to Array. Once the template has been defined, the main while loop is terminated and control moves on to frame 5. If the user enables 'Do not define template', for the first worm in the trap, which is the false condition for the case structure, then the template image matrix is padded with zeros and control moves onto frame 5. If the 'Do not define template' is enabled for the subsequent worms in the batch, then the template defined on the first worm continues to be used as the template image. The most recently defined template is always the one used for generating the correlation scores in a later portion of the code.

Frame 5 of main loop. This frame has a main case structure. If the worm is loaded, then the true Boolean value of the case is satisfied. The true case has two nested loops. The outer loop executes the inner loop twice. The number of times the inner loop runs is half the number of stacks specified by the user. During the first execution of the outer loop, the z stage moves n/2 number of stacks above the 'Initial focus z position' with an

increment of ‘Z distance between frames’ which is defined by the user and where n is number of stacks. In the second execution of the outer loop, the z stage moves n/2 number of stacks below the ‘Initial focus of z position’ in steps of the distance between the frames specified. If n is odd, then a correction is applied to get a valid value of n/2. The inner loop contains a stacked sequence structure whose first frame writes to the serial port of the z stage controller thereby making the z stage move incrementally depending upon the number of frames specified and the distance between frames. The second frame switches on the CFP fluorescence with a wait period of 300 ms. The live image is obtained and the fluorescence is turned off. The minimum background intensity is estimated form the pixel intensity values in the region defined as background in Frame 1 of the main loop. In the third frame, the maximum intensity of the pixels in the region enclosing the ASH, as defined by the user in Frame 1 of the main loop, is estimated. The live image is converted to a binary image using the IMAQ threshold VI. Two thresholds are applied, with the lower threshold being the mean of the background intensity the upper threshold being the maximum intensity. The pixels that lie between these thresholds are set to 1 and the rest to 0. The binary live image is then dilated and eroded using the IMAQ gray morphology VI. The grayscale live image and the segmented binary image are then multiplied using the IMAQ Multiply VI. The thresholded gray scale image and the template image are inputs to the MATLAB script window which contains the template matching code to generate the correlation scores. The correlation scores are calculated for each pixel location in the search image for every frame in the stack as previously described. The output of the MATLAB script window is the maximum correlation value in a matrix of correlation scores corresponding to every pixel location in

the search image matrix. If this value is greater than the threshold specified by the user, then the neuron is set to be detected and a bounding box is generated around the ASH neuron. If it is lower than the threshold specified, then the neuron does not get detected and the worm is flushed out as no bounding box is generated. The correlation scores generated and the z scan locations are stored in arrays. The two arrays are of the same size as every frame along the z axis has a corresponding maximum correlation score. The position of the z axis corresponding to the maximum correlation score is the best focus position for any given worm in the batch. There is a toggle button that enables the user to choose the edge detection method instead of the template matching method for younger worms. For this method, every z stage position corresponds to the Sobel edge threshold value instead of a correlation score and the frame with the highest threshold is considered to be the best focus frame. For the algorithm that combines both methods, the ten highest correlation scores are extracted and edge detection is performed on the search images corresponding to these ten scores. The frame with the highest edge threshold is then selected as the best focus position. The z stage moves to the best focus location at the end of the z scan using the serial port LabVIEW VISA VIs.

Frame 6 of the main loop. The CFP fluorescence is turned on and the neuron is subject to photobleaching with an exposure time specified by the user. If the ‘Manual cancellation of recording’ control is not enabled, the execution moves on to a stacked sequence structure. The first frame of this structure contains VIs that help specify the name and paths of the stored files. The ‘File number to start the recording from’ and the ‘Folder for storing data’ are specified by the user. The recorded frames are stored as ‘.tif’ images in the specified folder with the given file names. Recording takes place in the

next frame. The number of images acquired depends on the recording and the exposure time. After recording is completed, fluorescence is turned off. The files that were opened to save the recorded frames are closed in the next frame. There is a ‘Program Status’ indicator on the front panel that displays where the data have been saved. If the ‘Manual cancellation of recording’ control is enabled, photobleaching is stopped, no recording takes place and fluorescence is turned off.

Frame 7 of the main loop. Frame 7 contains a stacked sequence with 3 frames. In the first frame, the white light shutter is opened. The next frame contains a while loop and the difference in standard deviation between the background intensity and the intensity of the worm area is calculated. If this value is less than the ‘Threshold count’ specified by the user and the ‘Manual control pressure inlet’ control is disabled, valve lines 2, 5 and 7 are turned off. Otherwise, line 5 is enabled and the rest disabled. The worm is unloaded till the ‘Stop unloading’ control is selected or till the loop times out. There is an indicator on the front panel which confirms the worm’s exit. The next frame adjusts the position of the robotic arm for loading the next worm batch.

Frame 8 of the main loop. The valve control lines 2, 5 and 7 are disabled. If the ‘Stop the process’ control is selected, the camera is stopped and the VISA sessions to the robotic arm, z stage, shutter and FW are closed. The image frames are also deleted.
