# Supplementary material for: An automated compound screening for anti-aging effects on the function of C. elegans sensory neurons
Source: Sci Rep. 2017 Aug 24;7:9403. doi: 10.1038/s41598-017-09651-x (PMC5570957; doi:10.1038/s41598-017-09651-x)
Supplement: Supplementary file 2 — Supplementary Software 1 [file 41598_2017_9651_MOESM2_ESM.zip › Supplementary Software/Automated Recording/Tracking/Using Matlab/Instruction File.docx]

**Instruction for using the program for “Tracking the Neuron” in Matlab**

1. Copy the files named **‘corrMatching.m’** and **‘track.m’** and place them in a common folder.
2. Start Matlab and change the working directory path to the location where the files **‘corrMatching.m’** and **‘track.m’** are stored.
3. To track the neuron type the following command at Matlab prompt :

**>>** track(*‘path of the file to be tracked’*, *correlation score threshold*)

- Make sure that the path of the file to be tracked is enclosed within single quotation mark.
- The correlation score threshold is a numeric value and ranges from 0 to 1. A value of 0.7 should work well for most cases. If not, then try decreasing the threshold.

1. Hit enter after typing the track command.

- The first frame in the set of recorded images will appear in the **‘Maltab figure window’**. Carefully read the title of this figure window and enter the required information.
- Next, you will be asked to define a threshold for the neuron to be tracked. Define this threshold using the **‘Scale Bar’** shown on the bottom right of the figure window.
- After defining the threshold, press the **‘Tab’** shown on the bottom left of the figure window.
- A new figure window will appear on the screen which will show the neuron being tracked in the CFP /YFP channels and a plot of the **absolute FRET ratio** as a function of **Time(sec)**. The program assumes that an 80ms exposure time was used while recording the images.
- In case, while tracking, the program is unable to find the neuron then it will ask to redefine the neuron template. This might happen if the neuron is very dim.
- The program stores the CFP/YFP/FRET data into a file named **data.xlsx** in the current working directory, which will be the same as the folder where the files **‘corrMatching.m’** and **‘track.m’** are stored.
