# Supplementary material for: An automated compound screening for anti-aging effects on the function of C. elegans sensory neurons
Source: Sci Rep. 2017 Aug 24;7:9403. doi: 10.1038/s41598-017-09651-x (PMC5570957; doi:10.1038/s41598-017-09651-x)
Supplement: Supplementary file 2 — Supplementary Software 1 [file 41598_2017_9651_MOESM2_ESM.zip › Supplementary Software/Automated Recording/Advanced Display/ExTestAdvDispScaling.doc]

ExTestAdvDispScaling.vi

Select Tif file to load:

Hit white go arrow on top.

At top of display is a auto scale, zoom in, zoom out, and pin. Select Pin:

Now select Manual scale button, for Ratso Tif file select 500 for max and 0 for min. This allows user to set the min and max to scale the image to.

Now select histogram autoscale (this is similar to 5%-95%, and uses a histogram to how to scale the image, ignoring hot pixels and dead pixels).:

Now hit min-max (which uses the minimum value found in image and the maximum value found in image):

You can also right click in the image to get auto-scaling, pinning and zooming in and out:

Hit the stop button to end program.
